# A micro-CT-based method for quantitative brain lesion characterization and electrode localization

**DOI:** 10.1038/s41598-018-23247-z

**Published:** 2018-03-26

**Authors:** Javier Masís, David Mankus, Steffen B. E. Wolff, Grigori Guitchounts, Maximilian Joesch, David D. Cox

**Affiliations:** 1 Harvard University, Department of Molecular and Cellular Biology, Cambridge, MA 02138 USA; 2 Harvard University, Department of Organismic and Evolutionary Biology, Cambridge, MA 02138 USA; 3 Harvard University, Center for Brain Science, Cambridge, MA 02138 USA; 4 Institute of Science and Technology Austria, Klosterneuburg, Austria

## Abstract

Lesion verification and quantification is traditionally done via histological examination of sectioned brains, a time-consuming process that relies heavily on manual estimation. Such methods are particularly problematic in posterior cortical regions (*e*.*g*. visual cortex), where sectioning leads to significant damage and distortion of tissue. Even more challenging is the *post hoc* localization of micro-electrodes, which relies on the same techniques, suffers from similar drawbacks and requires even higher precision. Here, we propose a new, simple method for quantitative lesion characterization and electrode localization that is less labor-intensive and yields more detailed results than conventional methods. We leverage staining techniques standard in electron microscopy with the use of commodity micro-CT imaging. We stain whole rat and zebra finch brains in osmium tetroxide, embed these in resin and scan entire brains in a micro-CT machine. The scans result in 3D reconstructions of the brains with section thickness dependent on sample size (12–15 and 5–6 microns for rat and zebra finch respectively) that can be segmented manually or automatically. Because the method captures the entire intact brain volume, comparisons within and across studies are more tractable, and the extent of lesions and electrodes may be studied with higher accuracy than with current methods.

## Introduction

Many foundational discoveries in neuroscience have relied on observing changes in behavior following focal brain lesions. From the famous case of patient H.M., whose bilateral medial temporal lobe resection shed light on the role of hippocampus in learning and memory^1^, to more modern lesions in animal models^2,3^, focal brain lesions can play an important role in elucidating structure-function relationships in the brain.

In order to be maximally informative, lesion studies require precise spatially controlled lesioning procedures and techniques for exact quantification. The current gold standard for lesion quantification is to section the brain using a microtome, match the slices to an atlas, and then to report approximate coordinates of the largest and smallest lesions of all subjects in the study. The data to support the lesion experiment are frequently presented indirectly, as a set of camera lucida images or example histological slices^2–9^.

In addition, typical lesion quantification procedures are time-consuming and present many opportunities for failure: brain sections can warp, tear, stain unevenly, and degrade over time. Moreover, there is a loss of each section’s three-dimensional context in the brain. All of these factors make precise 3D reconstruction of a lesion in the brain challenging.

Another common use for lesions in neuroscience is to determine the precise location of micro-electrode recordings, by inducing small electrolytic lesions after the final recording session and later searching for them histologically. As with other lesion quantification applications, the requirement for sectioning and histology can make the process slow and imprecise. Electrolytic lesions are typically large relative to the electrode that produced them, but small enough that they are still challenging to locate in sections. As above, warping of tissue sections makes precisely reconstructing 3D arrangements of electrodes a challenge.

Here, we describe a new method for quantitative lesion characterization and electrode localization that leverages staining techniques common in electron microscopy (EM) with the power of x-ray micro-computed tomography (micro-CT). Micro-CT imaging is a technique in which a sample is placed in front of an x-ray beam path and rotated 360 degrees about a single, arbitrarily-chosen axis while a scintillator collects the transmitted x-rays not deflected by the sample^10^. This sequence of 2D projections of the sample is then used to reconstruct a three-dimensional volume using conventional computed-tomography reconstruction techniques. The end-product is a high-resolution 3D volume that can be digitally sliced in any orientation.

## Results

### Method Overview

**Figure 1:**
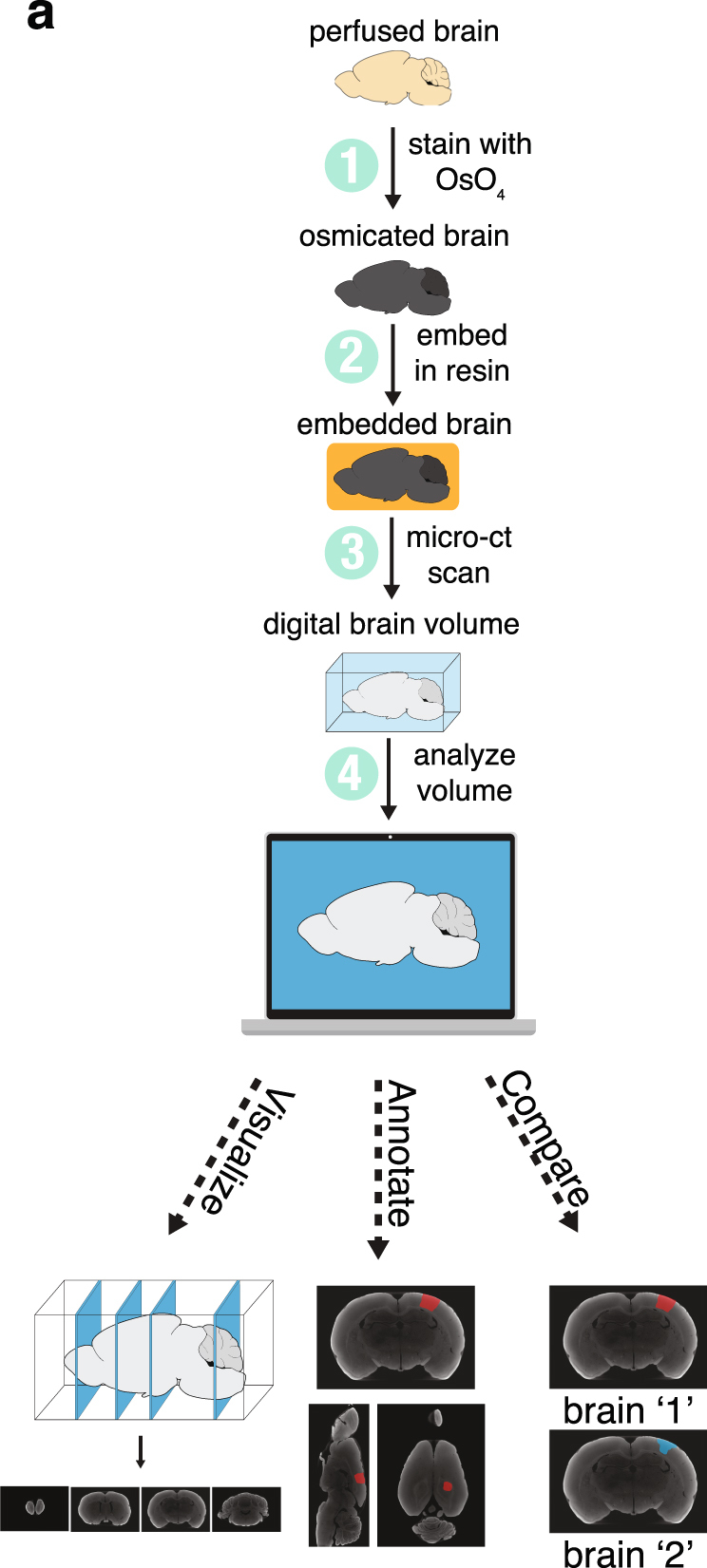
Method overview. (**a**) Overview of the steps required to prepare and analyze a whole brain using micro-CT imaging.

We developed a straightforward method for visualizing and analyzing small animal brains using micro-CT (Fig. 1). The conventional method involving sectioning, staining and light microscopy is error prone and labor-intensive, especially when multiple samples need to be analyzed. By relying on micro-CT imaging to analyze lesions using whole brains, the probability of damaging the sample and the amount of labor required to prepare the samples are greatly reduced. Moreover, this method lends itself to parallel processing of many samples, speeding up sample preparation considerably.

All care and experimental manipulation of animals were reviewed and approved by the Harvard Institutional Animal Care and Use Committee. First, we perfused the animal (a rat in this case) with paraformaldehyde (PFA) and glutaraldehyde (GA). Next, immediately after the perfusion, we incubated the sample for two weeks in osmium tetroxide (less time would be required for smaller brains). During this time, no interventions from the researcher are required.

Ensuring reliable penetration of osmium throughout the rat brain required some optimization of the protocol. We found that GA was an important addition to the perfusion protocol for ensuring reliable osmium penetration through the tissue (*see* Methods). We also found that using aqueous osmium (as opposed to osmium in a solution matched to tissue osmolarity such as sodium cacodylate) considerably improved osmium penetration deep into the brain (*see* Methods; Supplementary Fig. S1). As our method relies on mineralizing the brain with osmium, it is incompatible with any further histology or immunofluorescence of the same sample.

Recently, researchers have demonstrated whole mouse brain staining for electron microscopy using a method that alternates incubation in osmium and reduced osmium, combined with a number of specialized buffers^11,12^. This method was developed in order to preserve ultra-structure needed for electron microscopy imaging. We focused instead on developing a much simpler staining protocol that would allow the researcher to place the sample in one solution and revisit the sample when it was time for sample embedding.

After two weeks, we dehydrated the tissue and embedded the sample in resin. Because these steps only require solution exchanges, it takes roughly the same amount of time to process one sample as it does many. As a result, it is easy to process many brains in parallel.

Once the resin had cured, we placed the sample in a micro-CT machine and generated a 3D digital volume of the sample. Many institutions have in-house micro-CT machines, and commercial micro-CT scanning services are also broadly available, where researchers can ship samples (which are embedded in hard resin by this point) to the vendor and get 3D digital volumes back. Typical scanning of a sample with a micro-CT machine takes 30–60 minutes of preparation followed by a few hours of scan time that requires no user input (these imaging runs can also be done overnight). Once the digital volume is available, the researcher may visualize, segment and analyze the data with commercial or freely available software. A few of these packages include Avizo, VGStudio Max, SPIERS (free), Osirix (free), 3D Slicer (free), ImageJ (free), VAST (free) and Adobe Photoshop.

### Method Capabilities

**Figure 2:**
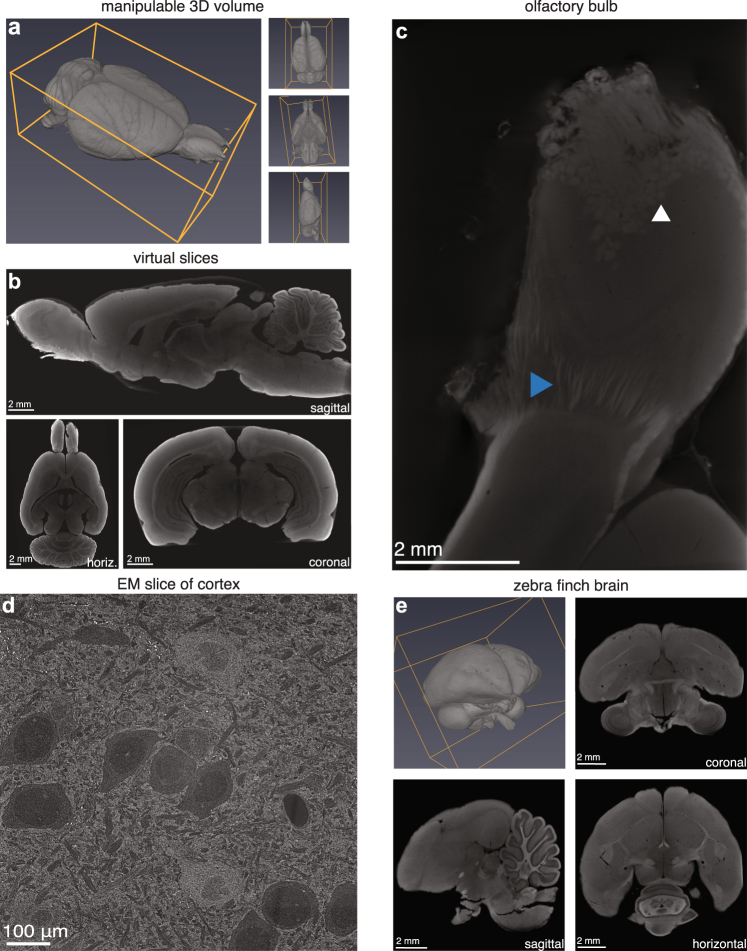
Capabilities of lesion characterization through micro-CT imaging. (**a**) Manipulable 3D volume and (**b**) virtual slices (13.9 μm voxels) at arbitrary orientations from a micro-CT scan of a Long-Evans rat. (**c**) Olfactory bulb scanned at higher resolution (4.9 μm voxels) revealing glomeruli (white arrowhead) and individual nerve fibers (blue arrowhead). (**d**) Scanning electron microscope (SEM) image (10 nm/pixel) of visual cortex of rat brain prepared for micro-CT imaging. (**e**) Zebra finch (*Taeniopygia guttata*) brain prepared for micro-CT imaging; 3D volume and 2D slices (5.6 μm voxels) in the coronal, horizontal and sagittal planes.

Employing micro-CT for lesion quantification enables high precision reconstruction without incurring substantial labor time (tissue preparation essentially consists of serially incubating the brain in different solutions) or cost (micro-CT machinery is available at many institutions and micro-CT scanning is available for modest fees via third party commercial services). The method results in a fully manipulable 3D volume (Video 1, Fig. 2a). Whereas with traditional sectioning one orientation must be chosen, with micro-CT scanning, the brain can be virtually sliced coronally, sagittally, horizontally, or at any arbitrary orientation (Fig. 2b). This means that lesions can be visualized and quantified from any plane.

The resolution of the scan depends on the size of the sample. For an entire rat brain, including the olfactory bulbs, we were able to achieve a nominal resolution of about 12–15 μm voxels, meaning that optical slices would have a thickness of 12–15 μm, considerably thinner than conventional physical slice thicknesses for lesion quantification. Smaller brains, such as whole mouse or zebra finch brains, may be scanned at higher resolution (~5.6 μm voxels, Fig. 2e). Subsets of the sample may also be scanned at higher resolution, in some cases revealing finer features, such as individual neural tracts and individual glomeruli in the olfactory bulb (4.9 μm voxels, Fig. 2c).

In order to ensure that our staining method faithfully preserved the tissue at the level of resolution required for micro-CT imaging, we re-purposed a brain prepared for micro-CT imaging, sliced a piece of its cortex into 30 nm sections and imaged it with a scanning electron microscope (SEM). We found that tissue features about an order of magnitude smaller than the micro-CT resolution were well-preserved, making us confident that our staining method was not producing gross damage or introducing artifacts into the sample (Fig. 2d). We note that while the stained tissue quality was adequate for micro-CT imaging, the protocol described here is not recommended for sample preparation for high-resolution electron microscopy, as ultra-structural damage is evident at the sub-micron scale.

In order to test the versatility of our staining method, we replicated the method with a zebra finch brain. We found that the procedure also stained the zebra finch brain satisfactorily, indicating that our staining protocol may be largely applicable to most small brains (Fig. 2e). While our method can in principle be applied to brains of all sizes, we predict a significant increase in necessary incubation time for larger brains, such as macaques.

### Brain Lesion Characterization

A major impetus for the development of this technique stemmed from attempting to quantify lesions in rat visual cortex. The visual cortex in the rat is in the far posterior end of the brain, and coronal sections in this area of cortex are not well attached to underlying subcortical structures. As a result, when sectioned, visual cortex often ends up in a few disconnected parts, further complicating attempts at 3D reconstruction. When these areas are lesioned, they make the already fragile sectioned tissue even more fragile, increasing the probability of damaging the sections during processing. By processing whole brains, we circumvent this problem by quantifying lesion extent in the context of an otherwise intact brain.

**Figure 3:**
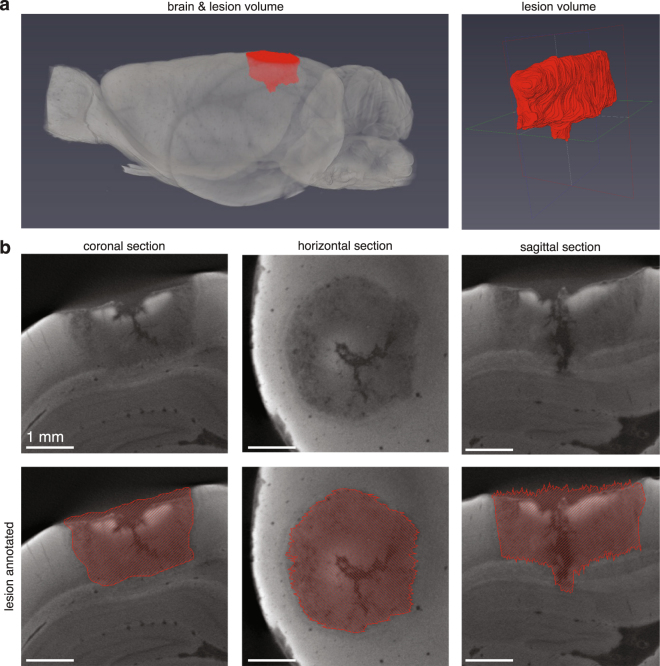
Visual cortex lesion characterization using micro-CT. (**a**) 3D visualization of rat brain lesioned in visual cortex with quinolinic acid. Left panel shows lesion volume in context of brain (brain made slightly transparent to allow visual access to lesion). Right panel shows isolated lesion volume. (**b**) 2D slices of lesion in the coronal, horizontal, and sagittal planes (12.8 μm voxels). Top three panels show the unsegmented sections; bottom three panels show sections with overlaid lesion annotation. This lesion was manually annotated in the coronal orientation every 2 slices. The volume was then created through interpolation.

We induced a lesion in the visual cortex of an adult rat, allowed the lesion to “set” for several days, and processed the brain as described above. After scanning, we manually segmented the lesion and generated a lesion volume (Fig. 3). Because digital volumes allow virtual slicing, we had the ability to quantify the lesion from any orientation. This is a big advantage over traditional sectioning where one orientation must be chosen at the time of sectioning because lesion boundaries may be more apparent in some views than others, and ambiguities can be resolved with much higher confidence (Fig. 3b). Further, because the brain is intact, we were able to visualize the lesion volume in context, a powerful tool for understanding the extent of damage and how it may correlate with behavior (Fig. 3a).

**Figure 4:**
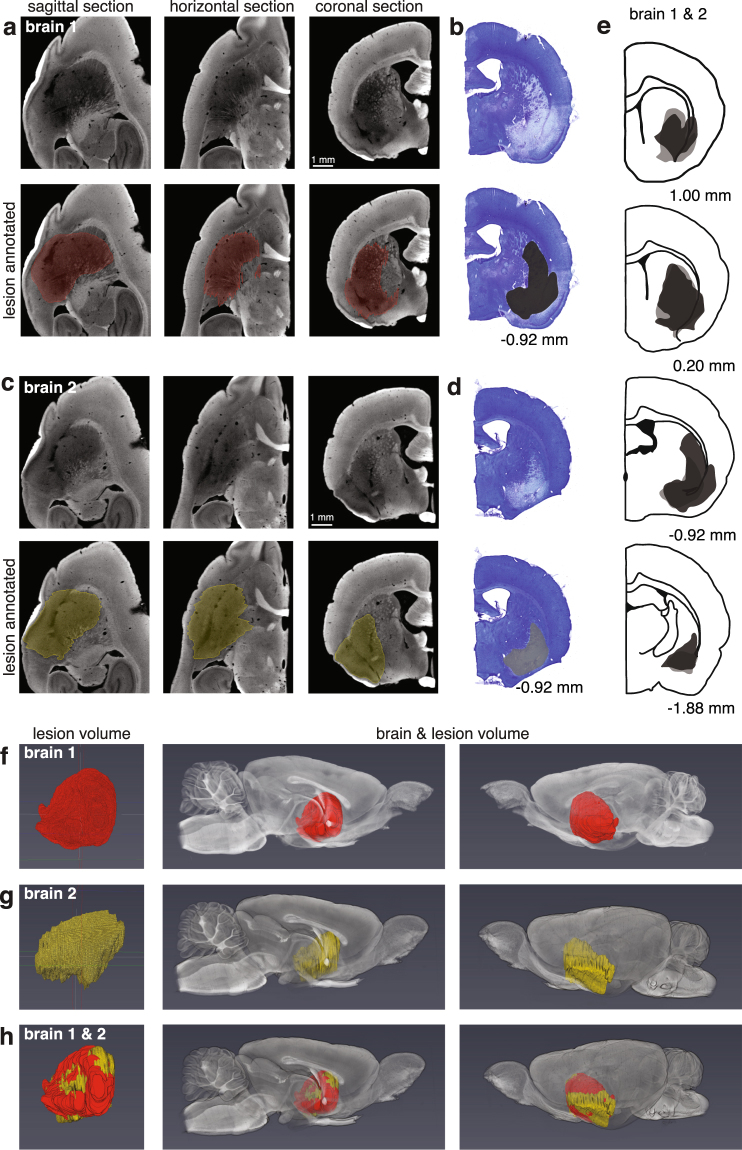
Comparison of dorso-lateral striatum lesion characterization using micro-CT and histology. (**a**) Sagittal, horizontal and coronal views of the left half of ‘brain 1’ processed for micro-CT imaging (13.5 μm voxels). Top three panels show unsegmented sections; bottom three panels show the annotated lesion on the same sections. Lesion manually segmented in sagittal plane every 2 sections and subsequently interpolated. (**b**) Closest matching coronal section of the right half of ‘brain 1’ processed histologically for light microscopy. (**c**,**d**) Are similar to (**a**,**b**) except for ‘brain 2’. Brain 2 lesion manually segmented in coronal plane every 8 sections. (**e**) Lesion characterization of histology-treated right halves of brains 1 (dark grey) and 2 (light grey). The numbers in (**b**,**d**,**e**) underneath the panels correspond to positions relative to bregma. (**f**) Lesion characterization of the left half of brain 1 processed for micro-CT. First panel shows the isolated lesion. Second and third panel show the lesion in context of the brain from two viewpoints. (**g**) Same as **f** for brain 2. (**h**) Overlay of the lesions in (**f**,**g**) illustrating capability for comparison of lesion characterization with digital brain volumes. The lesion in (**f**) was registered to the lesion in (**g**) and both are shown in the context of the left half of brain 2.

Having found that we could quantify surface cortical lesions well, we next sought to characterize deep lesions and compare these results directly with the results obtained from traditional sectioning. To this end, we induced large bilateral lesions in dorso-lateral striatum (DLS) and adjacent cortical regions in two animals. After extracting the brains, we separated the hemispheres and processed the right halves traditionally, and prepared the left halves for micro-CT imaging (Fig. 4). For the left hemisphere of brain 1, we decided to manually segment every other virtual slice (slice thickness = 13.5 μm, thus every 27 μm) in the sagittal orientation and then generate a lesion volume (Fig. 4a). For the left hemisphere of brain 2, we decided to manually segment every 8 slices in the coronal orientation (simulating ~100 μm thick slices) (Fig. 4c). The interpolation to generate the volumes from our annotation worked very well in both cases, as can be seen by the lesion extent in the orientations other than the ones manually segmented (Fig. 4a,c). This result indicates that researchers may prioritize detail or time when annotating lesions, both of which are advantages over traditional sectioning that normally results in time-consuming approximations.

We processed the right hemispheres traditionally, by slicing, Nissl-staining and imaging. Corresponding traditional and micro-CT slices are shown (Fig. 4b,d). In order to quantify lesion volume in the traditionally processed slices, we hand-registered the slices to the closest matching slice diagrams from an atlas^13^ and we multiplied approximate lesion areas with slice thickness to arrive at volume estimates.

Although the lesions on each hemisphere for each brain are different, we expected them to be similar given that we injected the same amount of toxin at the same depths. Propitiously, we found that our volume estimates through micro-CT and traditional methods were concordant (brain 1 left (micro-CT): 34 mm^3^, brain 1 right (traditional): 32 mm^3^; brain 2 left (micro-CT): 35 mm^3^, brain 2 right (traditional): 39 mm^3^).

Reporting the locations of lesions, and how consistent lesion are among different test subjects is important in lesion studies. Thus, we report lesion locations as done traditionally (Fig. 4e) and as possible through micro-CT preparation (Fig. 4f–h).

### Electrode Localization

Traditional methods for verifying electrode location in the brain involve many of the same histological methods as lesion quantification, and as a result, these methods suffer from many of the same drawbacks. Often, after recordings are completed, current will be sent down the electrode to induce an electrolytic lesion near the tip. Physiologists then look for this lesion after sectioning and staining the brain. A disadvantage of this approach is that the lesion is generally bigger than the tip of the electrode, introducing some uncertainty about where exactly it was located. In addition, during sectioning, the lesioned area may be challenging to find and precisely locate in the 3D context of the brain. Because conventional electrode materials have different x-ray absorption properties as compared to osmicated brain tissue, we next sought to test whether our method would allow *in situ* localization of electrodes in the brain.

**Figure 5:**
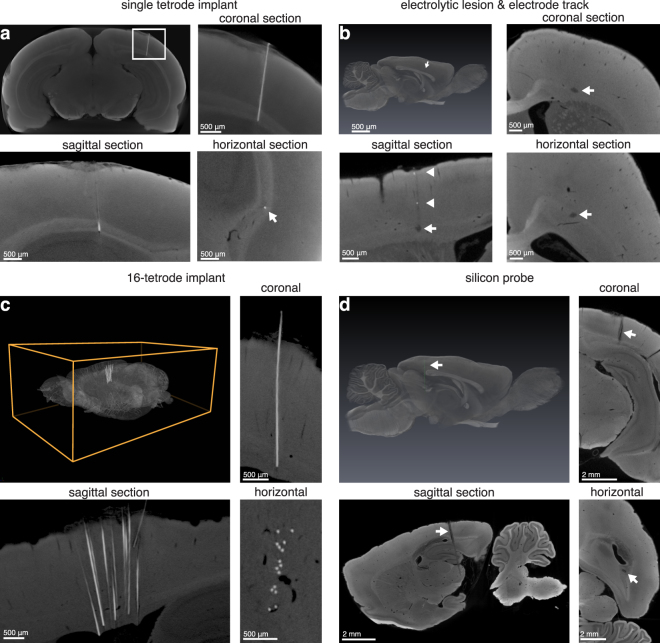
Electrode localization through micro-CT imaging. (**a**) Single tetrode left *in situ* (14.0 μm voxels). *Top left panel*: Max-intensity projection of virtual coronal sections showing location of nichrome tetrode traveling through visual cortex into the corpus callosum of a rat. *Top right*: Close-up of image shown in top left panel of (a) (white rectangle). *Bottom left*: sagittal and *Bottom right*: horizontal views of the implanted electrode (white arrow indicates location of the tetrode). (**b**) Electrolytic lesion and electrode track in anterior region of a rat brain (13.9 μm voxels). *Top left*: 3D rendering of brain with electrolytic lesion segmented out (white arrow indicating purple lesion). *Top right*: coronal, *Bottom left*: sagittal and *Bottom right*: horizontal sections indicating electrolytic lesion (white arrows) produced with a 75 μm diameter tungsten electrode. In addition, some metal deposited by the electrode upon retraction is visible along the track in the sagittal view (white arrowheads). (**c**) 16-tetrode implant left *in situ* in anterior cortex of a rat brain. *Top left*: 3D rendering of whole brain with 16-tetrode array left implanted. *Top right*: coronal, *Bottom left*: sagittal and *Bottom right*: horizontal sections indicating 16-tetrode implant (8.9 μm voxels). (**d**) Silicon probe (10 mm shank, 32 sites) left *in situ* traveling through posterior cortex, hippocampus and subcortical structures (13.9 μm voxels). *Top left*: 3D rendering of whole brain with segmented silicon probe (white arrow indicating green probe). *Top right*: Coronal, *Bottom left*: sagittal and *Bottom right*: horizontal views of silicon probe in the brain. Additionally, the reference site on the silicon probe is visible in the coronal and sagittal sections (tip of white arrows).

To test the utility of our method for localizing electrodes, we began by implanting a single tetrode in the brain of a rat and then subsequently perfused the animal with the electrodes in place. Recordings from this tetrode showed three isolated units (Supplementary Fig. S2). We perfused the animal, and prepared the brain as described above for lesion quantification. To reduce “ringing” artifacts associated with CT imaging of metal electrodes, we scanned with a metal filter in front of the x-ray source, filtering out the harder x-rays responsible for the ringing. Scan results show that the tetrodes were easy to find and could be visualized from any orientation (Fig. 5a).

Since it is common to implant multiple tetrodes at a time, we repeated the above procedure with a 16-tetrode array inserted into the anterior cortex of a rat. Post implantation, we severed the electrodes, leaving them in place. We then perfused the animal and prepared the brain for micro-CT imaging. The tetrodes were easily distinguishable in the resulting 3D volumes (Video 2, Fig. 5c). Interestingly, we found that many tetrodes experienced notable excursions as they penetrated the brain (Fig. 5). Despite this “drift” in the positions of the tetrodes, our method allowed unambiguous tracing from the tip of the tetrode to the connector at the skull.

While severing the electrodes and leaving them in place enables precise localization of multiple electrodes with our method, it may not always be desirable or possible to leave electrodes in the brain (e.g. in the case of acute recordings with stiff electrodes). We thus set out to test whether our method could also be used for the localization of electrolytic lesions, which at least addresses the challenges that come with sectioning and staining, even if localizing electrode positions this way still suffers from imprecision induced by the size of the electrolytic lesion. We implanted an acute recording tungsten electrode in the anterior cortex of a rat and passed a high current in order to induce an electrolytic lesion. The lesion “set” over the course of the next few days, and we perfused the animal and prepared the brain for micro-CT imaging. We found that the electrolytic lesion was readily apparent in our micro-CT dataset (Fig. 5b) with a volume of 0.0064 mm^3^ and a diameter of approximately 250 μm at its longest (it was oblong in shape).

We also asked whether our method could be used to locate the track of a retracted electrode. At a scanning resolution of 13.9 μm voxels, we were able to locate the track of a 75 mm diameter electrode (Fig. 5b *Bottom left* panel, white arrowhead), though the track was significantly more difficult to find as compared to the severed electrodes above.

Finally, while insulated metal electrodes are popular tools for neurophysiology, other recording technologies, such as silicon micro-machined electrode arrays are also widely used^14^. To test whether our method can also localize silicon probes, we implanted a rat with a silicon probe through posterior cortex, hippocampus and subcortical structures. We then severed the probe, allowed the animal to recover for 4 days (simulating conditions for chronic recording), and later perfused the animal and prepared the brain for micro-CT imaging. The silicon probe was easily identifiable in the resulting micro-CT volume (Video 3, Fig. 5d). Although the individual metal recording sites were not visible at our scanning resolution of 13.9 μm voxels (nor at a higher resolution scan of 7.4 μm voxels; data not shown), we did identify the probe’s larger reference electrode site higher up the silicon shank (Fig. 5d, *Top right* and *Bottom left* panels, white arrows). Because this site is a known distance from the silicon probe’s recording pads, the experimenter can nonetheless calculate the location of the probe’s recording sites. While the silicon probe itself appeared at a similar grey-level as the surrounding osmicated tissue, the probe track was nonetheless visible due to disruption of the tissue adjacent to the probe (*see* Video 3).

## Discussion

We have presented a new method for quantifying lesions and determining electrode location in small brains that combines electron microscopy-style staining techniques with micro-CT imaging. This method improves upon the quality of results of current standard methods, while at the same time requiring less labor and expertise to carry out. Although scientists have previously attempted to scan small animal brains with micro-CT machines (discussed below), the tissue preparation we have outlined here makes micro-CT imaging more accessible and useful for lesion quantification and electrode localization.

The method we propose comes at a time when there is increasing interest in micro-CT imaging and electron microscopy staining techniques. CT imaging has historically been common in the medical community (*e*.*g*. CT/CAT scans), but it is gaining popularity in biology. For example, micro-CT imaging is very useful to scientists in evolutionary and comparative biology, who employ the technique to generate 3D reconstructions of fossils, bones and exoskeletons^15^. Such samples are easy to scan because their chemical composition (*e*.*g*. the calcium carbonate in bones) makes them visible to x-rays without any prior treatment.

A key challenge for using micro-CT methods for brain imaging is that brain tissue is mostly transparent to x-rays. Fortunately, a variety of staining techniques exist that can adequately mineralize soft issue and overcome its contrast problem in a wide-range of samples, from fish to insects to squid hatchlings^16^. Thus, scientists who study soft tissue have increasingly been looking to micro-CT imaging. For example, developmental biologists have employed micro-CT imaging to track embryo development^17^, entomologists have employed it to study mating ethology in Drosophila^18^ and brain morphology in honeybees^19^, and very recently arachnologists have employed the method towards advancing spider neuroanatomy^20–23^.

Our work builds upon several previous studies that have suggested or applied micro-CT for imaging brains in various capacities^24^. For instance, one previous investigation performed on rabbits and mice proposed the use of micro-CT in the postmortem brain with a particular emphasis on its utility for histopathology, such as in the search for tumors and other abnormalities^25^. Although their samples were stained with proprietary MRI contrast agents, the authors predicted micro-CT’s potential usefulness in lesion studies as a time-saving, non-destructive alternative, as well as in genetic and drug discovery screens. Another study proposed staining mouse brains within the skull with iodine for quickly determining gross anatomical differences during a screen^26^. However, because of the use of iodine, the authors faced uneven shrinkage of the tissue and opted to embed it in a hydrogel to compensate for the change. Recently, scientists reported using micro-CT to assess cerebral cavernous malformations, a disorder resulting in abnormally large and irregular capillaries, in mouse cerebellum using both osmium tetroxide^27,28^ and aqueous iodine^29^. In the case of these studies, the samples were much smaller in volume (consisting of detached mouse hindbrains) and scanning resolution was reported at 9.5 μm voxels, whereas we reported higher resolutions for larger samples, such as whole zebra finch brains (scanned at 5.6 μm voxels). During our preliminary experiments, we found iodine potassium iodide solution (I_2_KI) did not satisfyingly stain nor generate the desired contrast in the rat brain. We did not observe any appreciable brain shrinkage using osmium and confirmed that the tissue was preserved at the cellular level (Fig. 2d).

Another alternative method for 3D brain volume reconstruction and lesion quantification is microscopic magnetic resonance imaging (*μ*MRI)^30^. There are several advantages to *μ*MRI, such as greater white/grey matter contrast^25^ compared to micro-CT and the ability to scan soft tissue *in vivo* (micro-CT has been shown to be safe *in vivo* for bone scans^31,32^). One study combined *μ*MRI, micro-CT and histology in order to create a brain atlas for the short-tailed opposum^33^. In the case of this study, however, the micro-CT imaging was used for the skull and the *μ*MRI was used for the brain. Nonetheless, the resolution of *μ*MRI is poorer (~25 μm voxels for similar samples^34^) and the technique is much more expensive because the machines are much more costly to obtain and maintain^25,35^. In many cases, *ex vivo* analysis of lesions is already a part of the standard work flow for lesion quantification, so the method proposed here represents only a mild modification to existing protocols.

Another possible method that could be implemented to quantify lesions in a similar fashion is high-resolution episcopic microscopy, similar to block-face EM, but with much thicker slices and optical microscopes^36^. However, this technique requires a specialized setup, does not have as much commercial support as micro-CT, and is typically geared more towards smaller samples, such as embryos. One could envision a block-face apparatus that allowed whole-brain sectioning, in which case such a technique might be as useful as the one proposed here. Disadvantages of such an approach include the need for specialized microscope and sectioning hardware and the fact that the sample is destroyed in the course of imaging.

Our work also extends upon previous efforts to use x-rays to localize electrodes in the brain^37–39^. While previous methods for x-ray electrode localization have relied on MRI for visualizing soft tissue, our method captures both the electrode and the surrounding tissue in a single volumetric image and at high resolution. We have demonstrated that our method allows for straightforward localization of single tetrodes, tetrode arrays, electrolytic lesions, electrode tracks and silicon probes.

Because our method produces rich 3D brain anatomy data, a valuable future direction would be to build a micro-CT “average brain” atlas from multiple co-registered brains, in order to standardize lesion location and size quantification. Expanding this method to a number of model organisms would also be worthwhile. Based on our results with a zebra finch brain (Fig. 2e), we also expect that extending this method to other species would only be a matter of determining incubation time in osmium based on brain size.

## Methods

### Surgeries and Electrophysiology

We conducted all animal experiments in accordance with NIH guidelines and protocols approved by the Institutional Animal Use and Care Committee (IACUC) at Harvard University. We used male and female Long-Evans rats (Charles River Laboratories, Wilmington, MA) for our experiments.

The general procedure for lesion experiments was to anesthetize animals with isoflurane, drill craniotomies and inject quinolinic acid (cat. no. #P63204, Sigma-Aldrich, St. Louis, MO) stereotaxically. We allowed the animals to recover for at least three days with appropriate analgesic treatment before perfusing them. To generate cortical lesions, we injected 200 nl of quinolinic acid (0.09 M, buffered to pH 7.3 in 1X PBS) at 100 nl/min at the following coordinates in mm relative to bregma (antero-posterior, medio-lateral, dorso-ventral): (−6.7, −4.0, −0.5), and (−6.7, −4.0, −1.5). These coordinates correspond to V1^13^. After each injection, we left the needle at the injection site for 5 minutes to allow for drug diffusion. In order to test the effectiveness of our method with deep brain lesions, we lesioned dorso-lateral striatum (DLS). To generate DLS lesions, we followed the same preparation as for cortical lesions, injecting 700 nl of quinolinic acid at the following coordinates relative to bregma: (−0.3, +3.6, −6.0), (−0.3, +3.6, −6.5), (+0.7, +3.6, −5.5) and (+0.7, +3.6, −6.0)^13^.

For electrophysiology experiments, we similarly anesthetized the animals, and drilled craniotomies. For the single tetrode implant, we implanted a tetrode stereotaxically in V1 and attached a head stage with dental cement. We drilled a second craniotomy in an anterior region of the contralateral hemisphere and inserted a ground wire parallel to the surface of the brain. We allowed the animal to recover overnight and recorded spontaneous neural activity the following day. We acquired electrical activity with a multiplexing headstage (RHD2132, Intan Technologies, Los Angeles, CA) at 30 kHz using Open Ephys software (http://www.open-ephys.org/gui/) and sorted the spikes with the [SPIKO]saurus [CLUST] MATLAB toolbox (https://github.com/jmarkow/spikoclust). Before perfusing the animal, we anesthetized it with isoflurane, and drilled off the head implant, severing the tetrode wire and leaving it still inserted in the brain. By leaving the wire in the brain, we aimed to maximize the accuracy in its localization. Moreover, because the tetrode is made of solid metal, we predicted it would scatter x-rays much more strongly than osmicated brain tissue, making it easy to locate.

For the 16-tetrode implant, we followed the procedure as described for a single tetrode but implanted the tetrodes in anterior cortex. As this experiment was a proof of concept, we did not conduct any recordings and sacrificed the animal after the surgery. Before sacrificing, we applied Vetbond (3 M, St. Paul, MN) to the electrodes and brain in order to prevent displacement and then cut the electrodes with scissors. An alternative method to sever the electrodes while introducing minimal disturbances would be to burn them off. Tetrodes were made by spinning together four 12.5 μm diameter nichrome wires (tetrode diameter approximately 25 μm) and gold-plating them to 250–400 kOhms. The tetrode array was made by threading tetrodes through an 8 × 2 grid of 34 AWG polyimide tubes (each tube had a diameter of 0.1601 mm, making each tetrode about 320 μm apart).

To induce an electrolytic lesion, we similarly anesthetized an animal and drilled a craniotomy in anterior cortex. We inserted a tungsten electrode (Scientific Microelectrode, Catalog #367-040605-00, Lot #30005086, Alpha Omega, Alpharetta, GA) with a 75 μm diameter and passed 40 μA of current for 15 seconds with the cathode to the animal’s tongue and the anode to the electrode. We retracted the electrode slowly and allowed the animal to recover and the lesion to set for 4 days.

To test whether silicon probes were detectable with our method, we implanted a 32-channel silicon probe with a 10 mm shank (A1x32, NeuroNexus, Ann Arbor, MI) into posterior cortex and through hippocampus of the same animal used to test electrolytic lesions. As this was a proof of concept, we did not record from this electrode and severed it with a pair of scissors, leaving it inserted in the brain. However, the electrode remained in the brain for several days, simulating the conditions of chronic recording, before the animal was perfused and the brain was prepared for imaging.

### Perfusion and Post-fixation

We anesthetized the animals in 4–5% isoflurane for 10–15 minutes to ensure full induction and then injected sodium pentobarbital (180 mg/kg; Fatal-Plus^®^ C IIN; #00298937368 Vortech Pharmaceuticals, Dearborn, MI) intraperitoneally. Once the animal was fully unconscious, we performed a high-pressure transcardial perfusion, first passing 2–400 ml 1X phosphate buffered saline (PBS) (depending on the size of the animal) at 300 mm Hg and then fixing with 2–400 ml 2% (w/v) paraformaldehyde (abbrev. PFA; #15710, Electron Microscopy Sciences (EMS), Hatfield, PA) and 2.5% (w/v) glutaraldehyde (abbrev: GA; #16220, EMS) in 1X PBS solution at 125 mm Hg. A video description of the incisions and brain extraction methods we employed can be found here^40^.

We incubated the brains in the same 2% PFA and 2.5% GA solution in 1X PBS at 4 °C while shaking lightly at 50 RPM on an orbital shaker for 48–72 hours. We made sure there was at least 10 times the volume of the brain in solution to ensure adequate post-fixation (in practice, this meant 40–50 ml of solution in a 50 ml conical centrifuge tube placed horizontally on the shaker).

We tried several variations of this perfusion and post-fixation protocol in an effort to get the osmium to diffuse evenly and completely throughout the entire brain. We found that the combination of PFA and GA (as opposed to just PFA) was important for consistent full osmium penetration of the tissue. Though we did not test it explicitly, we hypothesize this is because PFA fixation is reversible^41^, whereas glutaraldehyde fixation is not^42,43^. Given the two week incubation in osmium (*see* Staining and Embedding), it is possible that the PFA in the interior of the brain diffused out and the tissue degraded during the course of the staining, preventing the osmium from preserving the interior structures. The pressure of the perfusion (whether high-pressure, peristaltic or gravity-based) did not seem to have an effect on the quality of the staining based on our piloting, so we chose to carry out high-pressure perfusions because they were faster.

A previous study comparing glutaraldehyde and PFA in preparations of rat cerebral cortex for EM found that glutaraldehyde fixation for 24 hours was sufficient to protect the tissue and make it osmotically non-reactive^42^. At the nano-scale our tissue looks considerably damaged compared to the study, which we predict is because we perfuse with fixative buffered in 1X PBS whereas the study perfuses with glutaraldehyde buffered in sodium cacodylate. Thus, we predict an improvement to the tissue quality at the nano-scale could be achieved by perfusing and post-fixing with fixative buffered in sodium cacodylate, followed by an incubation in aqueous osmium. However, as we have mentioned, for micro-CT scanning, the tissue is well-enough preserved as we outline here, where we prioritized the simplicity of the procedure.

### Staining and Embedding

In finding an appropriate staining and embedding protocol, we aimed to achieve full, even osmium penetration throughout the entire rat brain while ensuring the protocol was as simple as possible.

Because rat brains are much larger than mouse brains, we faced considerable difficulty in finding a staining protocol that would achieve even osmium penetration throughout the entire rat brain. Previously, researchers have demonstrated even osmium penetration in mouse brains using a relatively complex protocol meant to preserve ultra-structure for electron microscopy^11,12^.

After an adequate post-fixation period, we washed the brains 3–4 times in ddH_2_O to remove any fixative and PBS. We then placed the brains in 40–50 ml of aqueous 2% OsO_4_ (#19190 EMS) in 50 ml conical centrifuge tubes. We sealed the tubes with Parafilm^®^ M (Bemis Company, Inc, Oshkosh, WI) and covered them completely in aluminum foil. We stored the brains at 4 °C shaking lightly at 50 RPM on an orbital shaker for two weeks (Supplementary Fig. S2). Horizontal placement of the tube on the shaker seemed to offer better mixing than vertical placement and may have improved osmium infiltration.

We found that the use of aqueous osmium (as opposed to osmium in a buffer like sodium cacodylate) was important for full tissue penetration. After numerous samples processed with osmium buffered with sodium cacodylate, we found that even after 6 weeks of incubation the osmium did not penetrate fully and seemed to be encountering a diffusion barrier about 2 mm into the tissue (Supplementary Fig. S2). Osmium was first described as a metal that dyed organic compounds^44^, and was later found to mostly bind to lipids in cell membranes^45–47^. If, for instance, there is a layer of intact cell membranes that is saturated with bound osmium, it could prevent further osmium from traveling deeper into the tissue because there are no passages for effective diffusion. Thus, we hypothesize that water as a solvent (and not sodium cacodylate) acts like a light detergent and permeabilizes the cell membranes enough so that even if a layer of cell membranes is saturated with osmium, there still exist paths through which unreacted osmium can continue to travel deeper into the tissue. A previous study also encountered a diffusion barrier, although it was microns not millimeters into the sample, and they were able to attribute it to precipitation in the extra-cellular space^11^.

After two weeks, we washed the brain in ddH_2_O, dehydrated it with ethanol dilutions and transferred it to pure acetone to prepare for resin infiltration and embedding. We allowed the resin (Durcupan™ ACM, #44610 Sigma-Aldrich, St. Louis, MO) to infiltrate the brain through resin/acetone dilutions and finally cured the brain in 22 × 40 × 20 mm polyethylene molds (Peel-A-Way^®^ Disposable Histology Molds, #27114, Ted Pella, Inc., Redding, CA) for two days at 60 °C. To prevent air bubbles leaving the sample during curing, we degassed the brains at 45 °C in a vacuum oven (Thermo-Scientific Lab-Line 6258 Vacuum Oven, Thermo Fisher Scientific, Inc., Waltham, MA) before curing. However, air bubbles should not affect the quality of the scan and this step is optional.

Detailed information on solutions, incubation times and temperatures can be found in Tables 1 and 2.

**Table 1:** Detailed Protocol for Small Whole-Brain Preparation for Micro-CT Scanning.

Step	Solution	Duration (min)	Temp. (C)
**Perfusion**			
Anesthesia	4–5% isoflurane gas	15	22
Lethal Injection	Sodium pentobarbital (180 mg/kg)	1	22
Reflex loss	Toe-pinch reflex exam	5–30	22
Blood removal	1X PBS	5–10	22
Fixation	2% (w/v) PFA, 2.5% (w/v) GA in 1X PBS	5–10	22
**Post-fixation**			
Post-fixation	2% (w/v) PFA, 2.5% (w/v) GA in 1X PBS (shaking gently)	2–3 days	4
Wash, 4 exchanges	ddH_2_O	1, 1, 1, 15	22
**Staining**			
Osmication	2% (w/v) OsO_4_ in ddH_2_O (sealed, wrapped, shaking gently)	2 weeks	4
**Embedding**			
Wash, 5 exchanges	ddH_2_O	1, 1, 1, 15, 60	22
Wash	ddH_2_O	30	4
Dehydration	20% (v/v) ethanol 80% (v/v) ddH_2_O	30	4
Dehydration	50% (v/v) ethanol 50% (v/v) ddH_2_O	30	4
Dehydration	70% (v/v) ethanol 30% (v/v) ddH_2_O	30	4
Dehydration	90% (v/v) ethanol 10% (v/v) ddH_2_O	30	4
Dehydration	100% ethanol	30	4
Infiltration	100% acetone	30	4
Infiltration	100% acetone	30	4
Infiltration	100% acetone	30	22
Infiltration	33% (v/v) Durcupan resin 67% acetone	3 hours	22
Infiltration	50% (v/v) Durcupan resin 50% acetone	3 hours	22
Infiltration	67% (v/v) Durcupan resin 33% acetone	3 hours	22
Infiltration	100% Durcupan resin	12 hours	22
Embedding	100% Durcupan resin	4 hours	22
Embedding	Degas in vacuum oven (non-essential)	15	45
Embedding	Cure in oven	48 hours	60
**Micro-CT**			
Scan	Micro-CT machine	4–5 hours	22
Reconstruct	Reconstruction software	15–30	22
Visualize/Analyze	2D/3D software (*e*.*g*. Avizo, Spiers, Osirix, FIJI, etc.)	30-hours	22

**Table 2:** Reagents for Small Whole-Brain Preparation for Micro-CT Scanning.

Purpose	Reagent	Catalog # and Vendor	Notes
Fixation	Paraformaldehyde (PFA)	#15710 Electron Microscopy Sciences (EMS)	2% (w/v) in 1X PBS
Fixation	Glutaraldehyde (GA)	#16220 EMS	2.5% (w/v) GA in 1X PBS
Osmication	OsO_4_	#19190 EMS	Work in fume hood
Dehydration	Ethanol	Koptec from Decon Labs	140, 190, 200 Proof
Infiltration	Acetone	#10015 EMS	Glass-distilled
Infiltration	Durcupan ACM resin	#44610 Sigma-Aldrich	A, B, C, and D components
Embedding	Disposable Molds	#27114 Ted Pella	Suggested

### Micro-CT Scanning

We scanned the samples at the Harvard Center for Nanoscale Systems using a Nikon Metrology X-Tek HMX ST 225 Micro-CT scanner (Nikon Metrology Ltd., Tring, UK), with a four megapixel detector (2000 × 2000 pixels), maximum voltage of 225 kV and maximum current output of 2000 μA. We used a molybdenum source metal (for the softest x-rays), scanned at 100 kV and 105 μA with a 1 second exposure time, and typically took 3,184 projections averaging 4 frames per projection. A 0.1 mm Cu filter substantially reduced the edge artifacts created by the edges of the brain. With the filter, settings were adjusted to 130 kV and 135 μA.

We reconstructed the samples (projections to z-stack) with CT Pro (Nikon Metrology Ltd., Tring, UK), the scanner software. We saved the data as z-stacks in TIFF format, making them easily readable by virtually any image processing software.

### Image Processing

We visualized the digital brain volumes with Avizo 9.1.1 (FEI, Hillsboro, Oregon) and VGStudio Max 3.0 (Volume Graphics, Heidelberg, Germany).

To annotate lesions, we created sub-volumes of the brains where the lesions were located and used a combination of the lasso and brush tools in Avizo to highlight lesioned areas. We used the fill holes and smoothing commands to regularize the edges and interiors of the masks. To illustrate the ability to annotate at different levels of detail, we annotated samples every 2 or every 8 virtual slices using different 2D views (*e*.*g*. coronal, sagittal). Once the entire lesions were outlined, we used the interpolation tool to generate full volumes. We calculated volumes by using the material statistics tool. We annotated the silicon probe and electrolytic lesion using the same methods in Avizo. In cases where we simply wanted to show different 2D views of the brain, we used VGStudio Max to generate coronal, sagittal and horizontal projections using the save image stack command.

Three-dimensional renderings of the brains were made with the volume rendering tool in Avizo and recorded with the snapshot tool. The 3D rendering of the brain in Fig. 5c was made in VGStudio Max by combining an image of the brain with low opacity and one with a high contrast threshold (excluding everything but the electrodes) in Photoshop to illustrate the electrode locations.

The brains typically only occupied a fraction of the image histogram, so to improve visualization we normalized (contrast-stretched) the pixel values by the boundaries of the darkest and lightest pixels present in the sample.

For our histological samples, we calculated lesion volumes by registering the sections to the closest-matching samples from an atlas^13^ (sections will typically distort in size/shape after histological treatment), multiplying lesion area per section by the section thickness (80 μm) and adding up the volumes calculated for every section.

The single tetrode in the brain in Fig. 5a was at a slight angle to the coronal plane, so to better visualize it in the coronal view, we created a maximum intensity projection of the slices that contained the electrode using FIJI^48^.

### Histology and Light Microscopy

For the samples present in Fig. 3, we cut the brains in half and processed one half for micro-CT as outlined above, and one half for histology and light microscopy.

We sectioned the brains into 80 μm sections using a vibratome. We mounted the sections onto slides and stained them with Cresyl-violet after serial ethanol dilutions. We imaged sections with an Axio Scan.Z1 (Zeiss, Oberkochen, Germany).

### Electron Microscopy

After micro-CT imaging, we bisected a brain sample with a bandsaw and machined a 7 mm × 7 mm × 20 mm block of cortex from one hemisphere. We mounted the block onto the chuck of a Leica EM UC-7 Ultramicrotome (Leica Microsystems, Buffalo Grove, IL), trimmed down the machining damage with a diamond knife and reduced the face to a 1 mm × 2 mm rectangle with a 400 μm setback. We then collected 30 nm sections onto carbon-coated Kapton tape with an automated tape-collecting ultramicrotome (ATUM)^49^. The tape with the sections was mounted to silicon wafers and we then post-stained the sections with 1% uranyl acetate in maleate buffer and 3% stabilized lead citrate (Ultrostain II, Leica Biosystems, Wetzlar, Germany). We imaged the sections on a Zeiss Sigma scanning electron microscope and acquired images using secondary electron detection.

## Electronic supplementary material

Video 1

Video 2

Video 3

Supplementary Information

## References

[1] Scoville W, Milner B (2000). Loss of recent memory after bilateral hippocampal lesions. 1957. J Neuropsychiatry Clin Neurosci.

[2] Kawai R (2015). Motor cortex is required for learning but not for executing a motor skill. Neuron.

[3] Otchy T (2015). Acute off-target effects of neural circuit manipulations. Nature.

[4] Wright N, Vann S, Aggleton J, Nelson A (2015). A critical role for the anterior thalamus in directing attention to task-relevant stimuli. J Neurosci.

[5] Kapgal V, Prem N, Hegde P, Laxmi T, Kutty B (2016). Long term exposure to combination paradigm of environmental enrichment, physical exercise and diet reverses the spatial memory deficits and restores hippocampal neurogenesis in ventral subicular lesioned rats. Neurobiol Learn Mem.

[6] Hosseini N, Alaei H, Reisi P, Radahmadi M (2017). The effects of NBM- lesion on synaptic plasticity in rats. Brain Res.

[7] Palagina G, Meyer J, Smirnakis S (2017). Complex visual motion representation in mouse area V1. J Neurosci.

[8] Ranjbar, H., Radahmadi, M., Reisi, P. & Alaei, H. Effects of electrical lesion of basolateral amygdala nucleus on rat anxiety-like behavior under acute, sub-chronic, and chronic stresses. *Clin Exp Pharmacol P*, 10.1111/1440-1681.12727 (2017). 10.1111/1440-1681.12727 28063155

[9] Wood, R. *et al*. The honeycomb maze provides a novel test to study hippocampal-dependent spatial navigation. *Nature*, 10.1038/nature25433 (2018). 10.1038/nature25433 PMC6342259 29364869

[10] Hsieh, J. *Computed Tomography*, *Second Edition*: *Principles*, *Design*, *Artifacts*, *and Recent Advances*, 2nd edn. (SPIEE Press, 2009).

[11] Mikula S, Binding J, Denk W (2012). Staining and embedding the whole mouse brain for electron microscopy. Nat Methods.

[12] Mikula S, Denk W (2015). High-resolution whole-brain staining for electron microscopic circuit reconstruction. Nat Methods.

[13] Paxinos, G. & Watson, C. *The Rat Brain in Stereotaxic Coordinates*, 4th edn. (Academic Press, Inc, 1998).

[14] Jun, J. *et al*. Fully integrated silicon probes for high-density recording of neural activity. *Nature* **551**, nature24636, 10.1038/nature24636 (2017). 10.1038/nature24636 PMC5955206 29120427

[15] Neues F, Epple M (2008). X-ray microcomputer tomography for the study of biomineralized endo- and exoskeletons of animals. Chem Rev.

[16] Metscher BD (2009). MicroCT for comparative morphology: simple staining methods allow high-contrast 3D imaging of diverse non-mineralized animal tissues. BMC Physiol.

[17] Johnson JT (2006). Virtual histology of transgenic mouse embryos for high-throughput phenotyping. PLoS Genet.

[18] Mattei AL, Riccio ML, Avila FW, Wolfner MF (2015). Integrated 3D view of postmating responses by the Drosophila melanogaster female reproductive tract, obtained by micro-computed tomography scanning. PNAS.

[19] Smith DB (2016). Exploring miniature insect brains using micro-CT scanning techniques. Sci Rep.

[20] Sombke A, Lipke E, Michalik P, Uhl G, Harzsch S (2015). Potential and limitations of X-Ray micro-computed tomography in arthropod neuroanatomy: A methodological and comparative survey. J Comp Neurol.

[21] Steinhoff P (2017). The synganglion of the jumping spider marpissa muscosa (Arachnida: salticidae): Insights from histology, immunohistochemistry and microCT analysis. Arthropod Struct Dev.

[22] Steinhoff P, Liedtke J, Sombke A, Schneider J, Uhl G (2017). Early environmental conditions affect the volume of higher-order brain centers in a jumping spider. J Zool.

[23] Stafstrom J, Michalik P, Hebets E (2017). Sensory system plasticity in a visually specialized, nocturnal spider. Sci Rep.

[24] Holdsworth D, Thornton M (2002). Micro-CT in small animal and specimen imaging. Trends Biotechnol.

[25] Crespigny A (2008). 3D micro-CT imaging of the postmortem brain. J Neurosci Methods.

[26] Anderson R, Maga A (2015). A novel procedure for rapid imaging of adult mouse brains with MicroCT using Iodine-Based contrast. PLoS One.

[27] Zhou Z (2016). Cerebral cavernous malformations arise from endothelial gain of MEKK3–KLF2/4 signalling. Nature.

[28] Choi J (2016). Micro-CT imaging reveals mekk3 heterozygosity prevents cerebral cavernous malformations in Ccm2-Deficient mice. PloS One.

[29] Choi, J., Yang, X., Foley, M., Wang, X. & Zheng, X. Induction and Micro-CT imaging of cerebral cavernous malformations in mouse model. *JoVE*, 10.3791/56476 (2017). 10.3791/56476 PMC5752174 28892037

[30] Benveniste H, Kim K, Zhang L, Johnson G (2000). Magnetic resonance microscopy of the C57BL mouse brain. Neuroimage.

[31] Brouwers J, van Rietbergen B, Huiskes R (2007). No effects of *in vivo* micro-CT radiation on structural parameters and bone marrow cells in proximal tibia of wistar rats detected after eight weekly scans. J Orthop Res.

[32] Klinck JR, Campbell GM, Boyd SK (2008). Radiation effects on bone architecture in mice and rats resulting from *in vivo* micro-computed tomography scanning. Medical Eng Phys.

[33] Majka, P. *et al*. A three-dimensional stereotaxic atlas of the gray short-tailed opossum (Monodelphis domestica) brain. *Brain Struct Funct* 1–17, 10.1007/s00429-017-1540-x (2017). 10.1007/s00429-017-1540-x PMC5884921 29214509

[34] Schneider JE (2003). High-resolution, high-throughput magnetic paragraph sign resonance imaging of mouse embryonic paragraph sign anatomy using a fast gradient-echo sequence. MAGMA.

[35] Sharpe J (2004). Optical projection tomography. Annu Rev Biomed Eng.

[36] Weninger WJ (2006). High-resolution episcopic microscopy: a rapid technique for high detailed 3D analysis of gene activity in the context of tissue architecture and morphology. Anat Embryol.

[37] Cox DD, Papanastassiou A, Oreper D, Andken B, James D (2008). High-Resolution Three-Dimensional microelectrode brain mapping using stereo microfocal x-ray imaging. J Neurophysiol.

[38] Borg, J. S. *et al*. Localization of metal electrodes in the intact rat brain using registration of 3D microcomputed tomography images to a magnetic resonance histology atlas. *eNeuro* **2**, 10.1523/ENEURO.0017-15.2015 (2015). 10.1523/ENEURO.0017-15.2015 PMC4550316 26322331

[39] Fu, T.-M. *et al*. Stable long-term chronic brain mapping at the single-neuron level. *Nat Methods* **13** (2016). 10.1038/nmeth.3969 27571550

[40] Gage, G. J., Kipke, D. R. & Shain, W. Whole animal perfusion fixation for rodents. *JoVE*, 10.3791/3564 (2012). 10.3791/3564 PMC3476408 22871843

[41] Helander, K. Kinetic studies of formaldehyde binding in tissue. *Biotechnic & Histochemistry*, 10.3109/10520299409106282 (1994). 10.3109/10520299409106282 8068812

[42] Paljärvi, L., Garcia, J. & Kalimo, H. The efficiency of aldehyde fixation for electron microscopy: stabilization of rat brain tissue to withstand osmotic stress. *Histochemical J*, 10.1007/BF01005026 (1979). 10.1007/BF01005026 110731

[43] Okuda, K., Urabe, I., Yamada, Y. & Okada, H. Reaction of glutaraldehyde with amino and thiol compounds. *J Fermentation and Bioengineering* **71** (1991).

[44] Tennant S (1804). On two metals, found in the black powder remaining after the solution of platina. Philos Trans Royal Soc.

[45] Bahr, G. Osmium tetroxide and ruthenium tetroxide and their reactions with biologically important substances: electron stains III. *Exp Cell Res* (1954). 10.1016/s0014-4827(54)80091-7 13220591

[46] Khan AA, Riemersma JC, Booij HL (1961). The reactions of osmium tetroxide with lipids and other compounds. J Histochem Cytochem.

[47] Riemersma, J. Osmium tetroxide fixation of lipids for electron microscopy a possible reaction mechanism. *Biochimica et Biophysica Acta* **152** (1968). 10.1016/0005-2760(68)90118-5 5660086

[48] Schindelin J, I A, Frise E, Kaynig V (2012). Fiji: an open-source platform for biological-image analysis. Nat Methods.

[49] Hayworth, K. J. *et al*. Imaging ATUM ultrathin section libraries with WaferMapper: a multi-scale approach to EM reconstruction of neural circuits. *Front Neural Circuits* 68, 10.3389/fncir.2014.00068 (2014). 10.3389/fncir.2014.00068 PMC4073626 25018701

