# Supplementary material for: A micro-CT-based method for quantitative brain lesion characterization and electrode localization
Source: Sci Rep. 2018 Mar 26;8:5184. doi: 10.1038/s41598-018-23247-z (PMC5980003; doi:10.1038/s41598-018-23247-z)
Supplement: Supplementary file 4 — Supplementary Information [file 41598_2018_23247_MOESM4_ESM.pdf]

Supplementary Information

A micro-CT-based method for quantitatve brain lesion characterization and electrode localization

Javier Masís[1,3,*], David Mankus[3], Steffen B.E. Wolff[2,3], Grigori Guitchounts[1,3], Maximilian Joesch[4], and David D. Cox[1,3]

[1]Harvard University, Department of Molecular and Cellular Biology, Cambridge, MA, 02138, USA
[2]Harvard University, Department of Organismic and Evolutionary Biology, Cambridge, MA, 02138, USA
[3]Harvard University, Center for Brain Science, Cambridge, MA, 02138, USA
[4]Institute of Science and Technology, Vienna, Austria
*jmasis@fas.harvard.edu

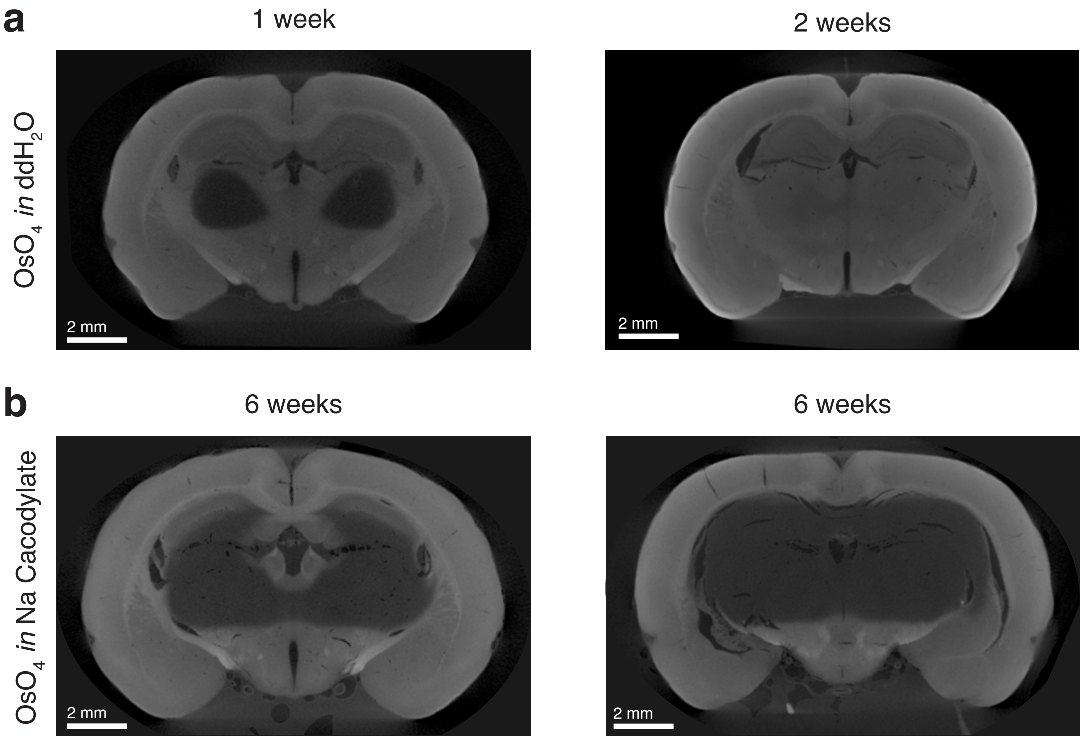

Figure S1: Effect of osmium tetroxide solvent on osmium penetration. (a) *Left panel*: Rat brain incubated in osmium tetroxide in ddH2O for 1 week. *Right panel*: Rat brain incubated in osmium tetroxide in ddH2O for 2 weeks. **(b)** Two rat brains incubated in osmium tetroxide in sodium cacodylate buffer for 6 weeks.

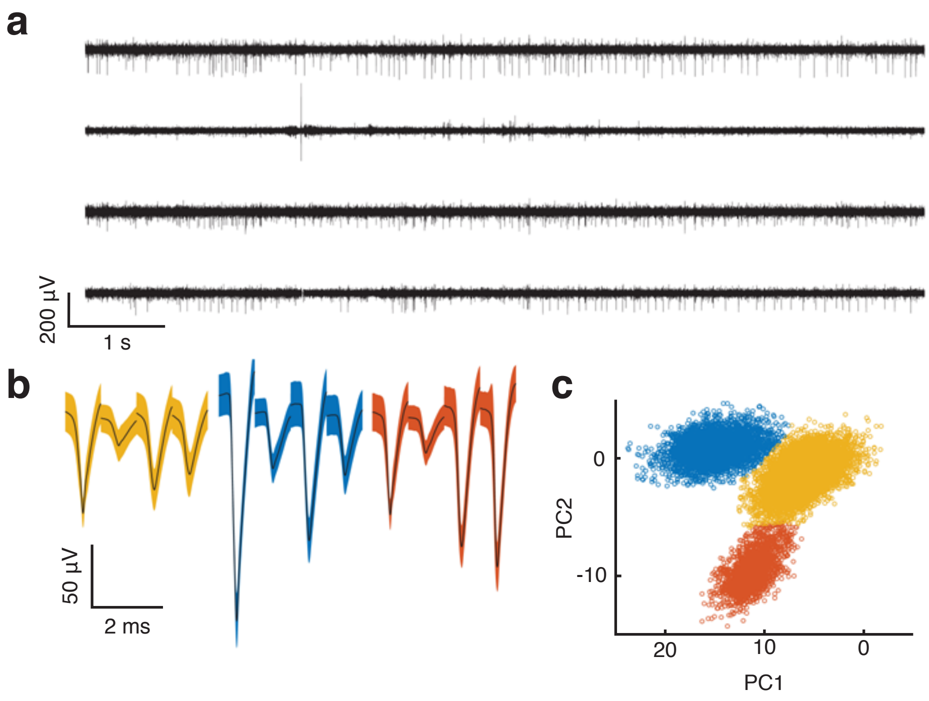

Figure S2: Units isolated from tetrode in Figure 5 (a) Example traces from four wires on a tetrode in visual cortex. **(b)** Sorted waveforms of three units. **(c)** First two principal components of sorted spikes.
